# Metagenomics and metatranscriptomics of prokaryotic and fungal microbiomes in produced water associated with petroleum degradation and pipeline corrosion from an oil terminal in Brazil

**DOI:** 10.1007/s11274-026-05012-x

**Published:** 2026-06-17

**Authors:** Rosimeire Floripes Gomes, Glen Jasper Yupanqui García, Mariana Santos Cardoso, Joyce da Cruz Ferraz Dutra, Vinicius de Abreu Waldow, Rubens Nobumoto Akamine, Maíra Paula de Sousa, Claudia Groposo, Bertram Brenig, Henrique Figueiredo, Vasco Ariston de Carvalho Azevedo, Aristóteles Góes-Neto

**Affiliations:** 1 Graduate Program in Microbiology, Federal University of Minas Gerais, Belo Horizonte, 31270-901 Brazil; 2 Graduate Program in Bioinformatics, Federal University of Minas Gerais, Belo Horizonte, 31270-901 Brazil; 3 Petrobras Research, Development and Innovation Center Leopoldo Américo Miguez de Mello, Petrobras, Rio de Janeiro, 21941-915 Brazil; 4 Petrobras, Criciúma, 88801-505 Brazil; 5 Institute of Veterinary Medicine, University of Göttingen Burckhardtweg, Göttingen 2, D-37077 Germany; 6 Veterinary School, Federal University of Minas Gerais, Belo Horizonte, 31270-901 Brazil; 7 Institute of Biological Sciences, Federal University of Minas Gerais, Belo Horizonte, 31270-901 Brazil

**Keywords:** Oil reservoirs microbial communities, Fungi, Hydrocarbon degradation, Pipeline corrosion, Meta-omics analyses

## Abstract

The prokaryotic microbial communities involved in hydrocarbon degradation and associated with oil pipeline corrosion have been extensively studied. Nonetheless, fungi can perform significant metabolic activities in these environments. Studies evaluating metabolically active microbial communities in oil reservoirs are limited. Our study investigated the total/DNA and active/RNA communities of Archaea, Bacteria, and Fungi in produced water samples from an onshore terminal in Brazil. DNA and RNA were sequenced using the Illumina HiSeq 2500 platform, and the meta-omics sequences were analyzed. Shannon alpha diversity (taxonomic and functional) revealed that total communities were more diverse than metabolically active ones, with Bacteria showing higher diversity than Archaea and Fungi. The bacterial genera *Syntrophotalea* (sulfur reducer) and *Pseudodesulfovibrio* (sulfate reducer) were most prominent in total communities, while *Halanaerobium* (acid producing) dominated active communities. These results confirm the presence of Microbially Influenced Corrosion (MIC); however, the aprAB and dsrABC genes showed very low expression. Methanogenic Archaea *Methanocalculus*, *Methanoplanus*, and *Methanothrix* were frequent in both total and active communities, and mcrABDG genes were significantly expressed in metatranscriptomic sequences. Fungal genera *Absidia*, *Penicillium*, and *Rhizopus* were dominant in DNA samples, whereas *Saccharomycodes*, *Pichia*, *Coemansia*, and *Schizosaccharomyces* dominated RNA samples. These fungi can remediate environments contaminated with recalcitrant hydrocarbons. Despite the limited information obtained from fungal functional profile, an in-depth investigation of their activities and interrelation with Archaea and Bacteria in oil reservoirs is crucial for monitoring and mitigating oil biodegradation and pipeline biocorrosion processes.

**Graphical abstract:**

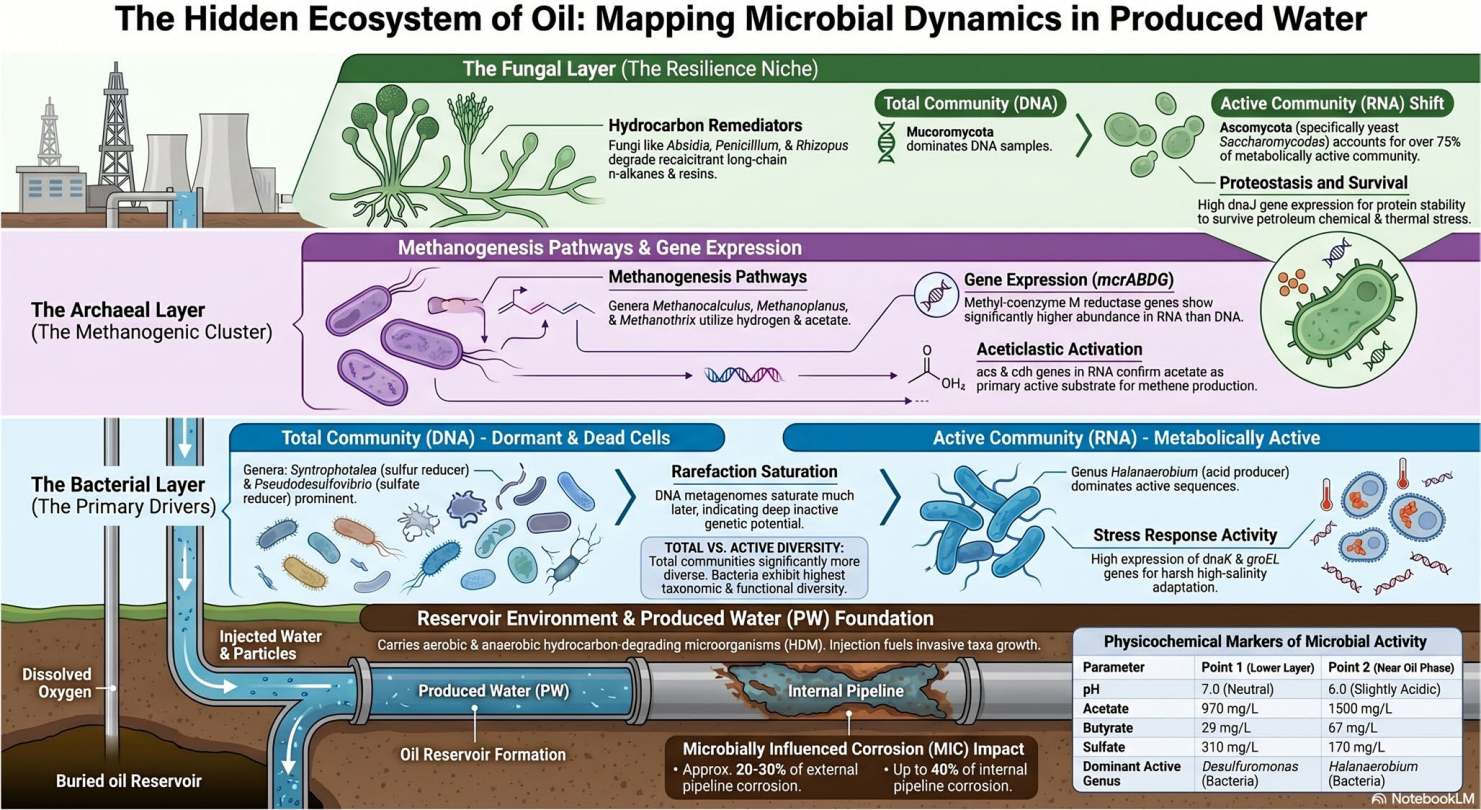

**Supplementary Information:**

The online version contains supplementary material available at 10.1007/s11274-026-05012-x.

## Introduction

Crude oil, a key component of the global energy supply, has attracted increasing attention in recent decades (Dong et al. 2019). The reservoir’s natural pressure is used during the initial oil production phase. Nonetheless, in the secondary recovery process, when oil reservoirs exhibit low pressure after years of activity, aqueous fluids, such as connate or fossil, fresh, marine, or mixed, can be injected into the reservoirs from injection wells, directing the oil towards the production wells located on the surface (Neff et al. 2011; Nazina et al. 2017; Liang et al. 2018; Salgar-Chaparro and Machuca 2019). Nonetheless, improper water injection favors the entry of suspended particles, microorganisms, or chemical impurities (Shestakova et al. 2011; Nazina et al. 2017). In addition, it significantly increases the volume of produced water (PW), the aqueous fraction of petroleum (Neff et al. 2011; Nazina et al. 2017).

PW can carry aerobic and anaerobic hydrocarbon-degrading microorganisms (HDM) (Shestakova et al. 2011; Nazina et al. 2017). Although oil reservoirs are considered anoxic environments, the injected water contains dissolved oxygen, promoting the proliferation of invasive aerobic taxa associated with the degradation of petroleum hydrocarbons (Shestakova et al. 2011; Nazina et al. 2017).

Bacteria are usually considered important in the biodegradation and mineralization of hydrocarbons due to their biochemical versatility (Pandolfo et al. 2023). Nevertheless, crude oil contains long-chain n-alkanes, polycyclic aromatic hydrocarbons, resins, and asphaltenes, which are recalcitrant to microbial degradation (Nzila and Musa 2021; Chand et al. 2022). Generally, Fungi have shown greater tolerance to harsh environments than Bacteria and can convert toxic compounds into less toxic or non-toxic end products; thereby, providing suitable conditions for Bacterial growth (Zhou et al. 2019). Moreover, Fungi can synthesize different cassettes of nonspecific intracellular enzymes (monooxygenases, nitroreductases, and cytochrome P450 transferases) and extracellular enzymes (laccases, hydrolases, and peroxidases) that allow them to degrade and utilize different hydrocarbons as sole carbon/energy sources (Gnanasekaran et al. 2019; Medaura et al. 2021; Rehman et al. 2021).

Besides HDM, aerobic and anaerobic Microbially-Influenced Corrosion (MIC) are also detected in PW (Nazina et al. 2017; Salgar-Chaparro and Machuca 2019). These microorganisms, usually Bacteria and Archaea, are classified according to their metabolic activities, such as sulfide-producing prokaryotes (including sulfate and thiosulfate reducers), acid producers, iron oxidizers and reducers, and methanogens (Nazina et al. 2017; Salgar-Chaparro and Machuca 2019). MIC contributes to the corrosion of pipeline surfaces, oil-gas-water separators, and tanks used for the passage and storage of fluids from oil production (Salgar-Chaparro and Machuca 2019). MIC is estimated to be responsible for almost 20–30% of external corrosion problems and about 40% of internal corrosion problems in pipelines (Wolodko et al. 2018).

In addition to Archaea and Bacteria, Fungi are known to synthesize metabolic products, including organic acids, from the biodegradation of hydrocarbons (Klug and Markovetz 1971; Porro and Branduardi 2017; Victor et al. 2020). These acids, when secreted in large quantities, can dissolve or selectively chelate copper, zinc, and iron from surfaces, making Fungi significant contributors to biocorrosion processes (Beech and Gaylarde 1999;Marangoni et al. (Marangoni, et al., 2013);Zhang et al. 2017). Additionally, Fungi can produce extracellular polymeric substances (EPS) that contribute to biofilm formation (Andreu and del Olmo 2023). Some microbial groups alter the electrochemical conditions at the metal/solution interface by attaching their cells, forming biofilms, and subsequently releasing metabolites, which induce or accelerate the corrosion process (Beech and Gaylarde 1999; Liu et al. 2023). Nevertheless, studies investigating the presence of fungal communities in oil field facilities remain scarce (Zhang et al. 2017); thereby, underestimating their contributions to biodegradation and biocorrosion processes in pipelines.

Currently, molecular high-throughput sequencing techniques have become crucial for obtaining genetic information from culturable and non-culturable microbial communities, as well as rare ones present in environmental samples, including those originating from oil reservoirs (Rachel and Gieg 2020; Zhou et al. 2022; Dutra et al. 2023). Thus, characterizing and investigating the metabolic activities of prokaryotic and fungal microbial communities, as well as their interrelationships in fluids resulting from oil production, are essential.

Therefore, the present study investigated the taxonomy and functional profiles of prokaryotic and fungal microbial communities in PW samples using DNA and RNA sequencing, high-throughput sequencing, and metagenomic and metatranscriptomic analyses.

## Materials and methods

### Collection, pre-processing, and preservation of samples

PW sampling was performed at an overland terminal for the transit and storage of oil in Duque de Caxias, Rio de Janeiro, RJ, Brazil (22° 42’ 52.88” S; 43° 17’ 13.29” W). The collection was performed in a drain tank, receiving residual oil and water fluids from other tanks and; therefore, comprising a representative set of all possible microorganism communities associated with this type of environment. This terminal was specifically selected for sampling due to prior monitoring by the oil company, which indicated the occurrence of MIC (Dutra et al. 2023), making it a relevant site for investigating microbial dynamics related to pipeline corrosion and oil degradation processes.

During sampling, the fluid had been resting for about 72 h, allowing the separation of the phases, with an aqueous lower layer and an oily upper layer, and; thus, there was no oil contamination in the aqueous phase. The samples were collected in clean, sterile plastic containers from two lateral valves connected to the tank at 1.00 m and 2.75 m, referred to as points p1 and p2, respectively. Before collection, the fluid standing in the outlet pipe of the valves was reinjected into the tank, and a new pumping occurred for acclimation. Three 5-liter containers of PW per sampling point (p1 and p2) were collected. At sampling, the fluids had an average temperature of 25 °C. The containers with the samples were transported at room temperature to the laboratory, about 25 km from the collection site. During transport and pre-processing of the samples, the temperature fluctuated by ± 2 °C.

The PW samples were immediately pre-processed in the laboratory using disposable and sterile vacuum filtration systems. This pre-processing method was previously developed in the study by Gomes et al. (2023). The systems contained a filter with a polyethersulfone (PES) membrane, with a diameter of 91 mm and a pore size of 0.2 μm (KASVI). Approximately 400 mL of PW was filtered from each collected container, totaling three filters per sampling point (p1 and p2). The filtration volume of 400 mL was standardized to allow comparability and replication. Before filtration, each container was inverted 10 times to ensure sample homogenization.

The filters containing retained biomass from the PW were preserved in RNAlater Stabilization Solution (Thermo Fisher Scientific), following the manufacturer’s instructions. After 24 h, the filters were stored in an ultrafreezer (-80 °C) until total DNA and total RNA extraction for metagenomic and metatranscriptomic analyses.

### Physicochemical characterization

According to Dutra et al. (2023), the physicochemical characterization of PW was carried out using biocorrosion diagnostic protocols for water and wastewater analysis (Clesceri et al. 2012). The analyses included pH, organic acids (lactate, acetate, propionate, formate, and butyrate) (Petrobras, 2020), sulfate, soluble sulfides, iron, chlorides, salinity, and thermal conductivity, all adapted according to Petrobras (2022a), together with alkalinity modified by Petrobras (Petrobras 2022b).

### Extraction, quantification, and quality of DNA and RNA

Four filters containing retained biomass from each sampling point (p1 and p2) were used for nucleic acid extraction, with two designated for DNA isolation and the other for RNA. Using sterilized stainless steel surgical blades, a quarter (1/4) of each filter was cut into small pieces and distributed equally into two tubes (technical replicates), yielding an amount sufficient for high-throughput shotgun sequencing. The remainder of the filter, three-quarters (3/4), were used in tests to optimize and validate DNA and RNA extraction protocols, as part of another study. Each replicate underwent separate extraction, library preparation, and sequencing, ensuring the integrity of biological variability in the analysis.

For DNA extraction, tubes were filled with 978 µL sodium phosphate and 122 µL MT Buffer, available on FastDNA™ SPIN Kit for Soil (MP Biomedicals). Tubes were filled with 1 mL of RNApro Soil Lysis Solution (MP Biomedicals) for RNA extraction. Those tubes also contained Lysing Matrix E (MP Biomedicals).

In order to facilitate the removal of biomass retained on the filters, the tubes were vortexed at moderate speed for 5 s and left to rest for 10 min. Subsequently, the tubes were homogenized in the FastPrep^®^ -24 Instrument (MP Biomedicals) at a constant speed of 6 m/s for 40 s. This homogenization step was repeated three times, incubating the tubes on ice for at least 2 min between each homogenization cycle to prevent overheating of the samples. The tubes were centrifuged at 14,000 × g for 10 min, and the supernatant was transferred to a new 2 mL tube to extract metagenomic DNA and metatranscriptomic RNA.

Metagenomic DNA was extracted from the supernatant using the FastDNA™ SPIN Kit for Soil (MP Biomedicals), applying the steps after cell lysis according to the manufacturer’s instructions. Quantification was performed on the NanoDrop2000 equipment (Thermo Fisher Scientific), and quality was assessed by 1% (w/v) agarose gel electrophoresis in 1X TAE.

For metranscriptomic RNA extraction, TRIzol reagent (Thermo Fisher Scientific) was added to the tube containing the supernatant in a 1:1 ratio. The mixture was incubated for 5 min to allow complete dissociation of the nucleoprotein complex. Subsequently, 0.2 mL of chloroform was added, and the mixture was incubated for 2 to 3 min. The mixture was centrifuged for 15 min at 12,000 × g at 4 °C, and the upper colorless aqueous phase, containing the total RNA, was transferred to a new tube. The RNA was precipitated at this stage by adding 0.5 mL of isopropanol (Sigma) and 10 µg of glycogen (Thermo Fisher Scientific). After these steps, the TRIzol protocol was continued according to the manufacturer’s instructions. Quantification was performed using the NanoDrop2000 equipment (Thermo Fisher Scientific), and quality was assessed by electrophoresis on a 1.2% (w/v) denaturing agarose gel, with the addition of 6% sodium hypochlorite in 1X TAE (Aranda, LaJoie and Jorcyk 2012).

### Sequencing of Metagenome and Metatranscriptome

Shotgun metagenomic sequencing was performed at Georg-August University Göttingen (Göttingen, Germany). The library was prepared from DNA sheared to approximately 450 bp of metagenomic DNA with the NEBNext Fast DNA Fragmentation and Library Preparation Kit (New England Biolabs), following the manufacturer’s instructions (Jotta et al. 2024). Library quality was assessed using Bioanalyzer 2100 (Agilent). For metatranscriptome sequencing, rRNA was depleted with the QIAseq FastSelect − 5 S/16S/23S Kit (Qiagen). The libraries were prepared using the NEBNext Ultra II Directional RNA Library Prep Kit for Illumina (New England Biolabs). Sequencing was performed using an Illumina HiSeq 2500. All metagenomic and metatranscriptomic sequences are available in the NCBI Sequence Read Archive (SRA) under accession number PRJNA1029995.

### Bioinformatics and statistical analysis

The metagenomes and metatranscriptomes were analyzed using the SqueezeMeta pipeline (Tamames and Puente-Sánchez 2020). This pipeline performs quality control of raw data, trimming adapters, and removing low-quality reads using the Trimmomatic tool based on standard parameters (Bolger et al. 2014). High-quality reads were assembled using the Megahit assembler (Li et al. 2015) in co-assembly mode. This mode merges all the metagenomes and configures them into a single one (forward and reverse) to improve the sequencing depth. The same protocol was applied to the metatranscriptome reads. After completing the assemblies, the pipeline separated the taxonomic and functional abundances by sample.

The Prodigal tool was used for gene prediction (Open Reading Frame - ORF) (Hyatt et al. 2010). The ORFs were aligned with the NCBI NR database for taxonomic assignments using the Diamond tool (Buchfink et al. 2015). For functional prediction, the ORFs were aligned with the latest public version of the KEGG databases (Release 114.0) (Kanehisa and Goto 2000). The taxonomic and functional abundance matrices generated in SqueezeMeta were extracted using the SQMtools package (Puente-Sánchez et al. 2020) in the R Software (version 4.2.0). The vegan v2.6.4 (Oksanen et al. 2019) and ggplot2 v3.5.2 (Wickham 2016) packages were used for the analysis of alpha and beta diversity at the genus and orthologous gene (KEGG ID) levels.

Alpha diversity of the total (DNA) and metabolically active (RNA) microbial communities from PW samples at points p1 and p2 was assessed using the Shannon, Chao1, Simpson, and Evenness indices. These indices were calculated using PAST software v. 4.04. Normality of the data was verified using the Shapiro-Wilk test, and statistically significant differences in alpha diversity values and relative abundances at the genus level among microbial domains (Archaea, Bacteria, and Fungi) were evaluated using ANOVA, followed by Tukey’s post-hoc test for multiple comparisons. For non-normally distributed data, the Kruskal-Wallis test followed by Dunn’s post-hoc test was applied.

Venn diagrams were prepared using the Venn package (v1.11) (https://github.com/dusadrian/venn) to compare the shared and unique genera between the samples. The principal coordinates analysis (PCoA), using the Bray-Curtis distance, was performed with the vegan package to visualize the beta diversity of archaeal, bacterial and fungal microbial communities, and the plots were generated with the ggplot2 and ggrepel (v0.9.5) (Slowikowski et al. 2018) packages, and the permutational multivariate analysis of variance (PERMANOVA, 9999 permutations, *p* < 0.05) was employed to analyze the dissimilarity of microbial communities, using PAST v. 4.04 (REF).

We also used NMDS (Non-Metric Dimensional Scaling), a flexible, assumption-free analysis, to analyze our complex multivariate community-ecology data. In NMDS, there is no assumption about taxon response shape, unlike RDA, which assumes linear responses, and CCA, which assumes unimodal responses; real data often violate both assumptions. Furthermore, NMDS works well with non-normal, zero-inflated, and heterogeneous ecological data, while RDA/CCA can be distorted by outliers or non-linearities. All data were previously Hellinger-transformed (which converts taxon abundance data to the square roots of relative abundances), and Bray-Curtis dissimilarity was used as the resemblance measure.

The matrices of the relative abundances of the 100 most abundant orthologous genes (KEGG ID) identified in the metagenomic and metatranscriptomic sequences of the PW samples from points p1 and p2 were analyzed with the STAMP software (v2.1.3) (Parks et al. 2014). STAMP verified the significant differences in the orthologs between the analyzed samples using the ANOVA test (*p* < 0.05) with the Tukey-Kramer as post-hoc test (confidence level 0.95), and the effect size by Eta-squared. Based on those analyses, heatmaps of the predicted functional profiles were generated.

## Results

### Physicochemical characterization

**Fig. 1:**
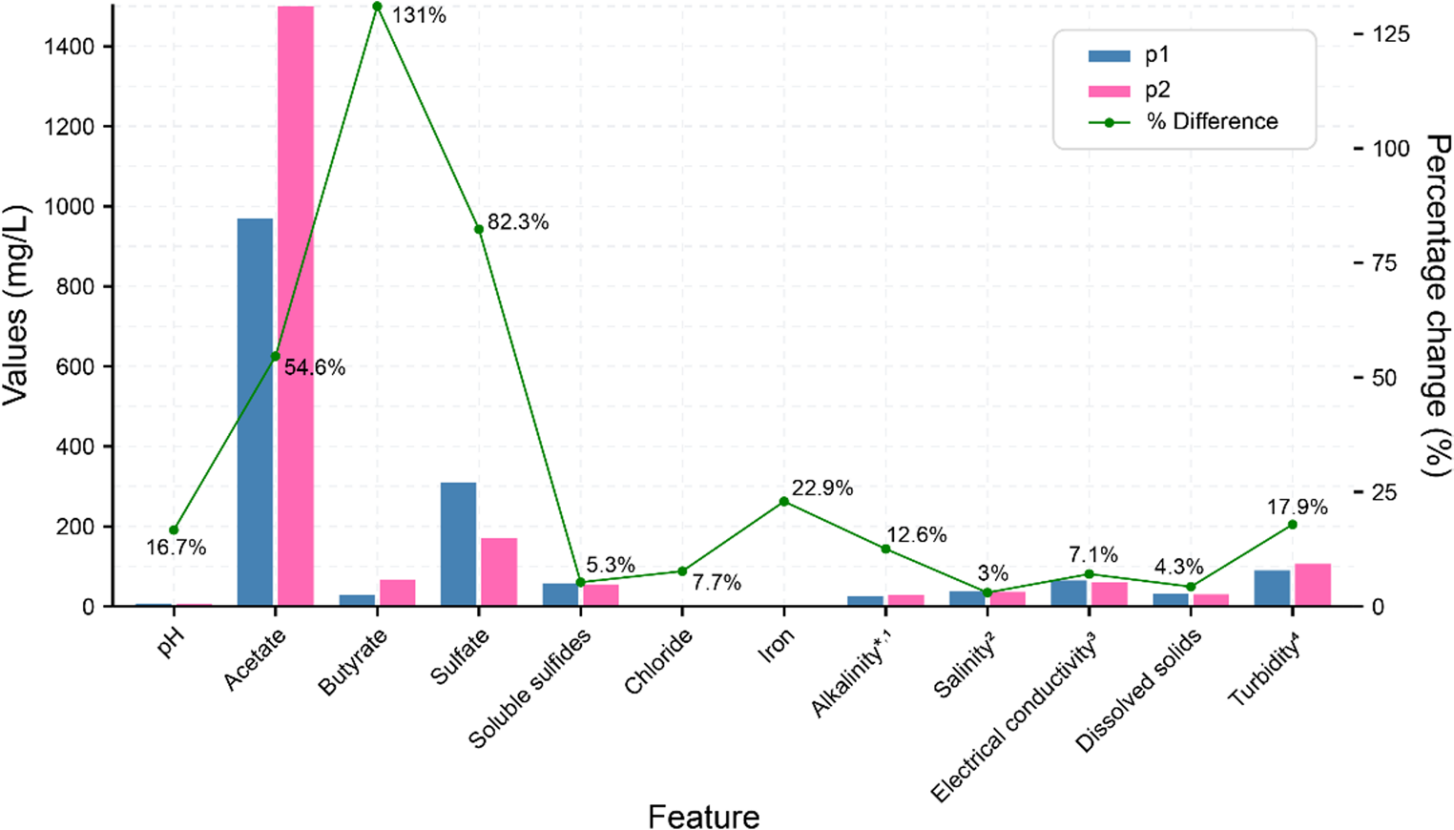
Physicochemical characterization of produced water samples collected at points p1 and p2 from the drainage tank. (Dutra et al. 2023). Sampling points: drain tank valve connected at 1.00 m height (p1) and 2.75 m height (p2). nd: not detectable. *1st inflection: pH = 3.2 (p1), pH = 3.0 (p2) Unit: ^1^milliequivalents per liter (megs/L); ^2^parts per thousand (ppt); ^3^Milisiemens (mS); ^**4**^Nephelometric turbidity unit (NTU)

Characterization of the PW samples collected at points p1 and p2 revealed variations in the analyzed parameters (Fig. 1, Supplementary Material 1). pH showed a percentage variation of 16.67%, with values of 7.0 and 6.0 at points p1 and p2, respectively (Fig. 1). Acetate exhibited a variation of 54.64%, with concentrations of 970 mg/L (p1) and 1500 mg/L (p2), while butyrate showed the greatest percentage difference among the analyzed compounds, reaching 131.03%, with concentrations of 29 mg/L (p1) and 67 mg/L (p2). Sulfate content varied by 82.35%, with values of 310 mg/L (p1) and 170 mg/L (p2). Soluble sulfides showed a variation of 5.28% (Fig. 1).

Among inorganic ions, chloride presented a percentage difference of 7.69%, and soluble iron varied by 22.92%, with concentrations of 0.48 mg/L (p1) and 0.59 mg/L (p2). Alkalinity varied by 12.55%, salinity by 3.02%, electrical conductivity by 7.07%, and dissolved solids by 4.30% (Fig. 1). Finally, turbidity showed a variation of 17.89%, with values of 90.13 NTU (p1) and 106.25 NTU (p2) (Fig. 1).

These variations in physicochemical properties suggest potential differences in microbial communities at points p1 and p2. To explore this, total DNA and RNA were extracted from the samples, followed by metagenomic and metatranscriptomic sequencing to perform detailed analyses.

## DNA and RNA extraction and sequencing

Nucleic acids, DNA and RNA, were extracted from the PW biomass retained on filters corresponding to the sampling points (p1 and p2) (Supplementary Material 2). To assess technical variability, DNA and RNA were isolated from two biological replicates, each with two technical replicates, performed in duplicate. For DNA, the average yields were 20,912 ± 2,116 ng (p1) and 24,200 ± 1,736 ng (p2), with mean A260/280 ratios of 1.85 and 1.84, and A260/230 ratios of 0.23 and 0.36, respectively (Supplementary Material 2). For RNA, the average yields were higher, reaching 41,354 ± 7,134 ng (p1) and 45,884 ± 21,512 ng (p2), with mean A260/280 ratios of 1.81 and 1.77, and A260/230 ratios of 0.48 and 0.43, in that order (Supplementary Material 2). After extraction, high-throughput sequencing and subsequent metagenomic and metatranscriptomic analyses were performed.

DNA sequencing generated 338,806,536 reads (p1) and 341,463,352 reads (p2). For RNA, the number of reads ranged from 53,097,210 (p1) to 48,325,692 (p2). All reads were of high quality. Following the assembly process, 334,440,687 reads (98.7%) (p1) and 337,336,078 reads (98.8%) (p2) were successfully mapped to contigs based on DNA sequences. For RNA, 51,327,944 reads (96.7%) (p1) and 47,013,730 reads (97.3%) (p2) were mapped. These high-quality, mapped sequences enabled comprehensive analyses, including the assessment of alpha diversity of microbial communities, characterization of taxonomic composition and abundance, analysis of beta diversity and community similarity, as well as functional profiling of microbial assemblages.

### Alpha diversity of PW microbial communities

**Fig. 2:**
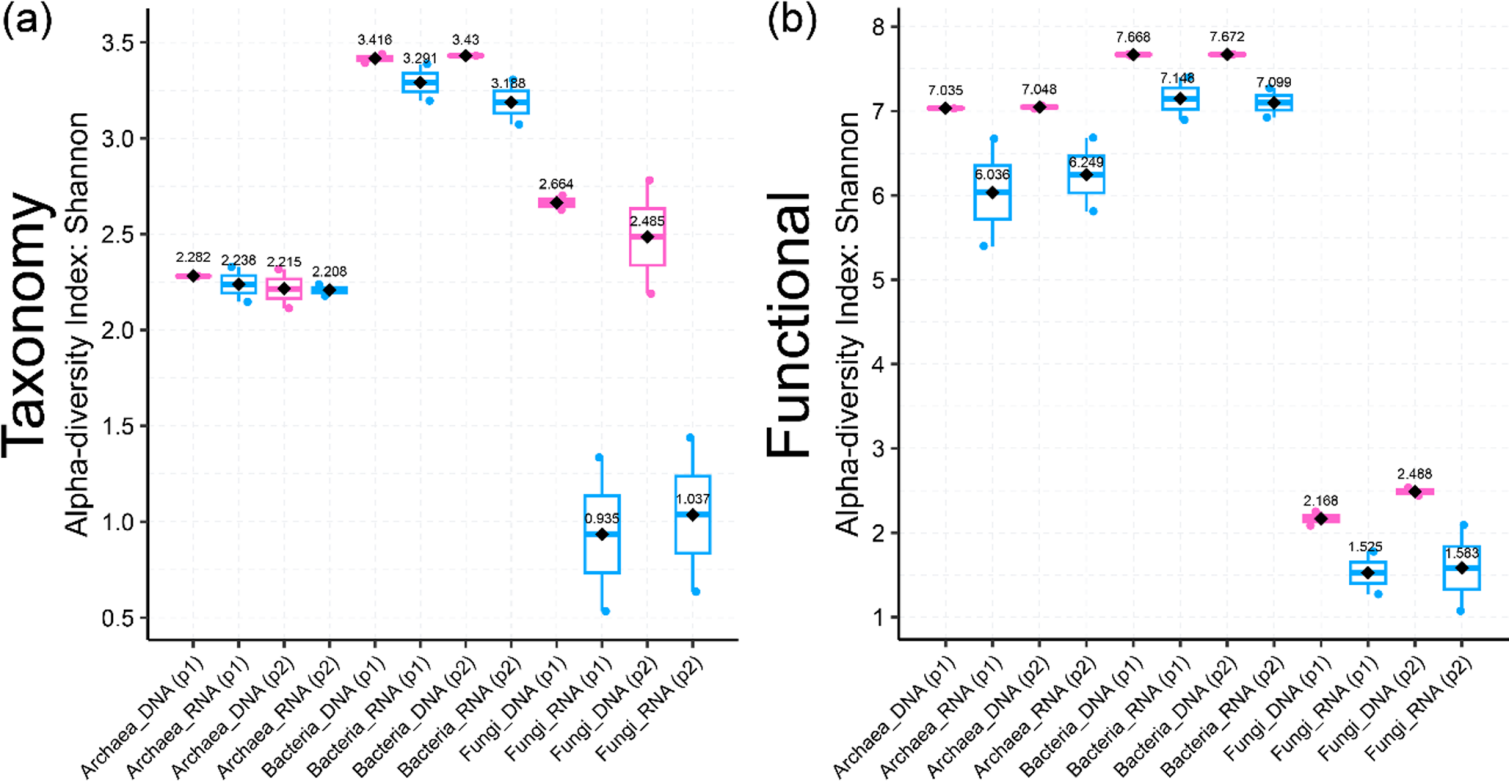
Analysis of (**a**) taxonomic and (**b**) functional alpha diversity, at the genus level, of the total/DNA and metabolically active/RNA communities of (**a**) Archaea, (**b**) Bacteria, and (**c**) Fungi from produced water samples from points p1 and p2. Sampling points: drain tank valve connected at 1.00 m height (p1) and 2.75 m height (p2). Note: Analyses were performed from biological replicates (BR) 1 and 2, each consisting of two technical replicates (TR)

Metagenomic and metatranscriptomic sequences obtained from PW samples from points p1 and p2, at the genus level, was used to assess the taxonomic and functional profile of the total/DNA and metabolically active/RNA microbial communities of Archaea, Bacteria, and Fungi (Fig. 2, Supplementary Material 3).

Based on the metagenomic sequences, the total microbial communities (DNA) showed statistically significant differences among the domains Archaea, Bacteria, and Fungi (Fig. 2, Supplementary Material 3), as revealed by analysis of variance (ANOVA), for the taxonomic Shannon diversity index (p1: *p* = 0.00016; p2: *p* = 0.03504), functional Shannon diversity (p1: *p* = 0.00749; p2: *p* = 0.00207), Chao1 richness (p1: *p* < 0.0001; p2: *p* < 0.0001), Evenness (p1: *p* = 0.001088), and Simpson diversity (p1: *p* = 0.003159). At point p2; however, Evenness and Simpson indices did not differ significantly among domains. Multiple comparisons using Tukey’s test showed that total Bacterial communities exhibited significantly higher taxonomic diversity (p1: *p* = 0.00007) and functional diversity (p1: *p* = 0.00538; p2: *p* = 0.00137). At point p2, based on the taxonomic Shannon index, a statistically significant difference was observed between Bacteria and Archaea (*p* = 0.03543), whereas no significant differences were found between Archaea and Fungi.

Based on the metatranscriptomic sequences, statistically significant differences were also identified in the diversity of metabolically active/RNA microbial communities of Archaea, Bacteria, and Fungi (Fig. 2, Supplementary Material 3). ANOVA revealed differences among domains for taxonomic Shannon diversity (p1: *p* = 0.01475; p2: *p* = 0.01868), functional Shannon diversity (p1: *p* = 0.00499; p2: *p* = 0.004301), and Chao1 richness (p1: *p* = 0.001547; p2: *p* = 0.01163). In contrast, the Evenness and Simpson indices did not show significant differences among microbial groups. Tukey’s test revealed that active Bacterial communities had significantly higher taxonomic diversity compared to fungal communities (p1: *p* = 0.01308; p2: *p* = 0.01655), as well as higher functional diversity compared to both Archaeal (p1: *p* = 0.009794; p2: *p* = 0.007623) and fungal (p1: *p* = 0.00519; p2: *p* = 0.004697) communities. Additionally, the Chao1 index indicated greater richness in Bacteria compared to Archaea (p1: *p* = 0.00254; p2: *p* = 0.01852) and Fungi (p1: *p* = 0.00181; p2: *p* = 0.01331).

Using the t-test, no significant differences were observed between the total/DNA and metabolically active/ RNA communities at sampling point p1 (Archaea: *p* = 0.71476; Bacteria: *p* = 0.33350; Fungi: *p* = 0.15808) or p2 (Archaea: *p* = 0.93347; Bacteria: *p* = 0.28614), except for Fungi at point p2, which showed a statistically significant difference (*p* = 0.04652). Similarly, no significant differences were detected in the comparison between points p1 and p2 within the DNA (Archaea: *p* = 0.63152; Bacteria: *p* = 0.65190; Fungi: *p* = 0.61386) or RNA fractions (Archaea: *p* = 0.70902; Bacteria: *p* = 0.12926; Fungi: *p* = 0.91913).

**Fig. 3:**
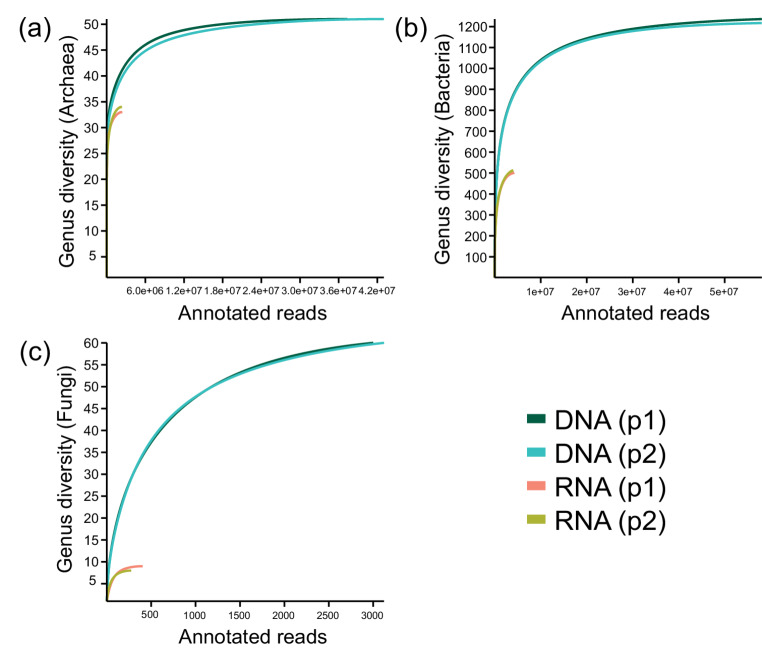
Rarefaction curves based on annotated genera for total/DNA and metabolically active/RNA microbial communities of (**a**) Archaea, (**b**) Bacteria, and (**c**) Fungi from produced water samples collected at points p1 and p2. Sampling points: drain tank valve connected at 1.00 m height (p1) and 2.75 m height (p2). Analyses were performed from biological replicates (BR) 1 and 2, each consisting of two technical replicates (TR)

Although the statistical comparisons revealed few significant differences, the diversity profiles became more evident when assessed through rarefaction curves (Fig. 3), which provided complementary insights into community structure. Based on the annotated genera, the curves revealed greater diversity in the DNA metagenomes compared to the RNA metatranscriptomes for Archaea, Bacteria, and Fungi, with curve stabilization indicating saturation of the detected diversity, especially for the DNA samples (Fig. 3).

### Composition of PW microbial communities

**Fig. 4:**
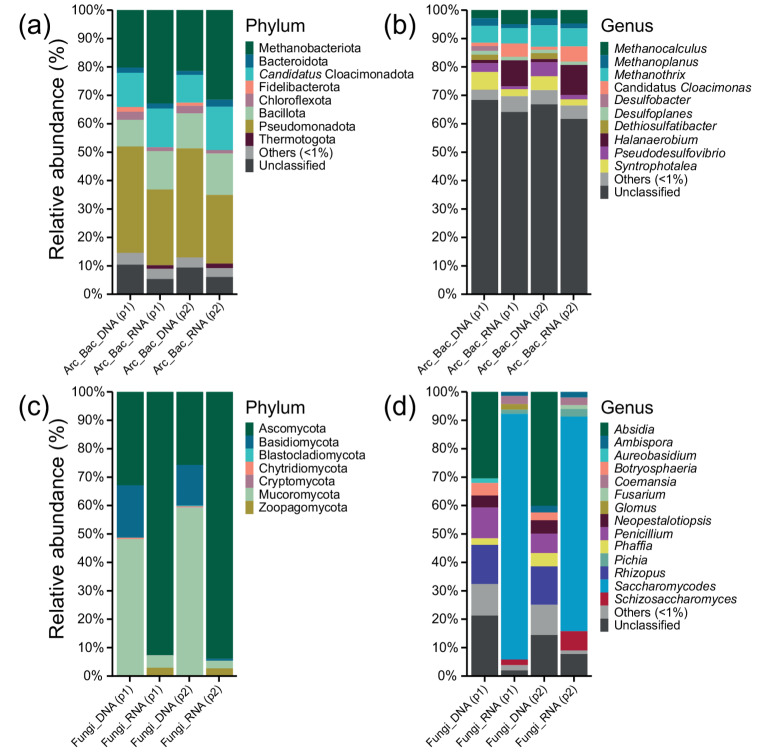
Relative abundance, at the phylum and genus levels, of the total/DNA and metabolically active/RNA microbial communities of (**a** and **b**) Archaea and Bacteria, and (**c** and **d**) fungus from produced water samples from points p1 and p2. Sampling points: drain tank valve connected at 1.00 m height (p1) and 2.75 m height (p2). Analyses were performed from biological replicates (BR) 1 and 2, each consisting of two technical replicates (TR). Abundances less than 1% were grouped and named “Other.” “Unclassified” microorganisms in the database were also unified

At the phylum and genus level, the relative abundance of the total/DNA and metabolically active/DNA microbial communities from points p1 and p2 of PW were evaluated (Fig. 4, Supplementary Material 4). Abundances under 1% were grouped and named “Other” for better visualization. Similarly, the “Unclassified” microorganisms in the database were also unified (Fig. 4, Supplementary Material 4).

The phylum Methanobacteriota, belonging to the Archaea domain, showed an abundance of over 20% in all evaluated samples (Fig. 4, Supplementary Material 4). The genera of Archaea *Methanocalculus*, *Methanoplanus*, and *Methanothrix* were the most representative among PW’s total and metabolically active microbial communities from points p1 and p2 (Fig. 4, Supplementary Material 4). When comparing the abundances obtained in all samples, no significant difference was observed between the results of Archaea (Kruskal-Wallis, *p* = 0.852).

The phyla Pseudomonadota, Bacillota, and “Candidatus” Cloacimonetes, belonging to the Bacteria domain, were the most abundant (Fig. 4, Supplementary Material 4). *Syntrophotalea* and *Pseudodesulfovibrio* were the most representative in the total Bacterial communities, showing abundances over 4% and 3%, respectively (Fig. 4, Supplementary Material 4). In contrast, the genera *Halanaerobium* and “Candidatus” Cloacimonas were the most abundant among PW’s metabolically active Bacterial communities from points p1 and p2 (Fig. 4, Supplementary Material 4). Statistical analyses confirmed the existence of a significant difference between the abundances of DNA and RNA samples from both points (Dunn’s post hoc p1, *p* = 8.380 × 10^− 5^; p2 = 1.550 × 10^− 4^).

In the analysis of fungal communities, based on DNA sequences, the phylum Mucoromycota showed abundances over 47% (Fig. 4, Supplementary Material 4). Nonetheless, in the analysis of RNA sequences, the phylum Ascomycota was the most representative, exhibiting abundances over 92% (Fig. 4, Supplementary Material 4). Among the total fungal communities, the genus *Absidia* was dominant (40%), followed by the genera *Rhizopus*, *Penicillium*, and *Neopestalotiopsis* (Fig. 4, Supplementary Material 4). Nevertheless, the genus *Saccharomycodes* was the most represented among the metabolically active communities, exhibiting abundances over 75% (Fig. 4, Supplementary Material 4). The genera *Coemansia*, *Schizosaccharomyces*, *Pichia*, *Ambispora*, *Glomus*, and *Fusarium* were also identified as active communities in the PW samples from points p1 and p2 (Fig. 4, Supplementary Material 4). For the results of Fungi, similarly to the results of Bacteria, statistical analyses confirmed the existence of a significant difference between the abundances of DNA and RNA samples from both points (Dunn’s post hoc p1, *p* = 5.292 × 10^− 11^; p2 = 3.297 × 10^− 4^).

**Fig. 5:**
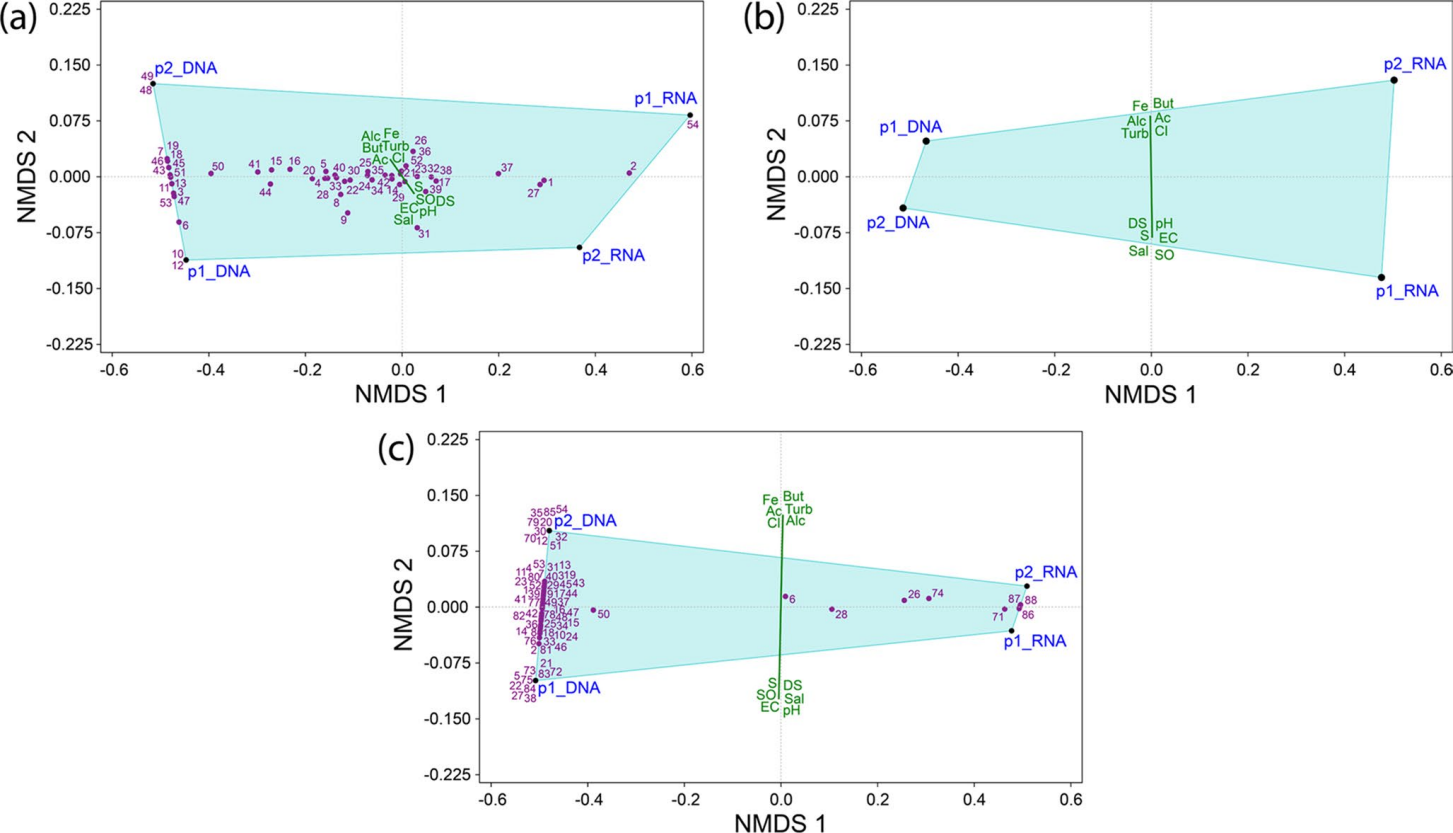
Non-Metric Dimensional Scaling (NMDS) of the total/DNA and metabolically active/RNA microbial communities of (**a**) Archaea, (**b**) Bacteria, and (**c**) Fungi from produced water samples at points p1 and p2. Sampling points: drain tank valve at 1.00 m (p1) and 2.75 m (p2). The analysis was performed using all the genera and 12 environmental variables. Note: The points corresponding to the genera of microorganisms were only depicted in (**a**) Archaea and (**c**) Fungi, as in (**b**) Bacteria, there are 1372 genera, and showing all of them would compromise the analysis of the general patterns observed

Non-Metric Dimensional Scaling (NMDS) to analyze the total and active microbial communities using all the genera from each domain and the environmental variables of produced water (PW) at p1 and p2 (Fig. 5). The interpretations presented below should be regarded as exploratory only.

For Archaea, the active microbial communities at p1 and p2 clustered near the vectors for pH, SO₄²⁻, S²⁻, salinity, EC, and DS, and were associated with methanogenic genera such as *Methanocorpusculum*, *Methanoregula*, and *Methanocalculus*, as well as *Methanoplanus* and *Methanolobus* (Fig. 5). In contrast, the total communities at p1 and p2 were positioned near the vectors for acetate, alkalinity, butyrate, Cl⁻, Fe, and turbidity, correlating with other methanogenic genera, including *Methanothrix*, *Methanothermobacter*, and *Methanohalophilus*, and *Methanococcus* (Fig. 5).

The bacterial communities at p1 clustered with pH, SO₄²⁻, S²⁻, salinity, EC, and DS, and were associated with sulfate-reducing and fermentative genera such as *Desulfobacter*, *Syntrophotalea*, *Sphaerochaeta*, and *Mesotoga* (total/DNA), and *Desulfuromonas* (active/RNA) (Fig. 5). By contrast, the communities at p2 were more strongly related to acetate, alkalinity, butyrate, Cl⁻, Fe, and turbidity, with prominence of sulfate-reducing, hydrocarbon-degrading, and syntrophic genera such as *Pseudodesulfovibrio*, *Geotoga*, and “Candidatus” *Cloacimonas* (total/DNA), as well as *Oceanidesulfovibrio*, *Paucidesulfovibrio*, and *Halanaerobium* (active/RNA) (Fig. 5).

Similar to the Archaea and Bacteria, the fungal communities, including decomposers and fermentative yeasts, showed associations with the environmental variables evaluated. At p1, the communities clustered with the vectors for pH, SO₄²⁻, S²⁻, salinity, EC, and DS, and were primarily represented by the genera *Penicillium*, *Botryosphaeria*, *Rhizopus*, *Glomus*, *Saccharomycodes*, and *Coemansia* (active/RNA), and *Pyrrhoderma* and *Rhizophagus* (total/DNA) (Fig. 5). At p2, the communities were closer to acetate, alkalinity, butyrate, Cl⁻, Fe, and turbidity, with *Pichia* and *Schizosaccharomyces* standing out (active/RNA), and *Ambispora*, *Hyphopichia*, *Absidia*, and *Podila* (total/DNA) (Fig. 5).

### Beta diversity and Similarity of PW Microbial Communities

**Fig. 6:**
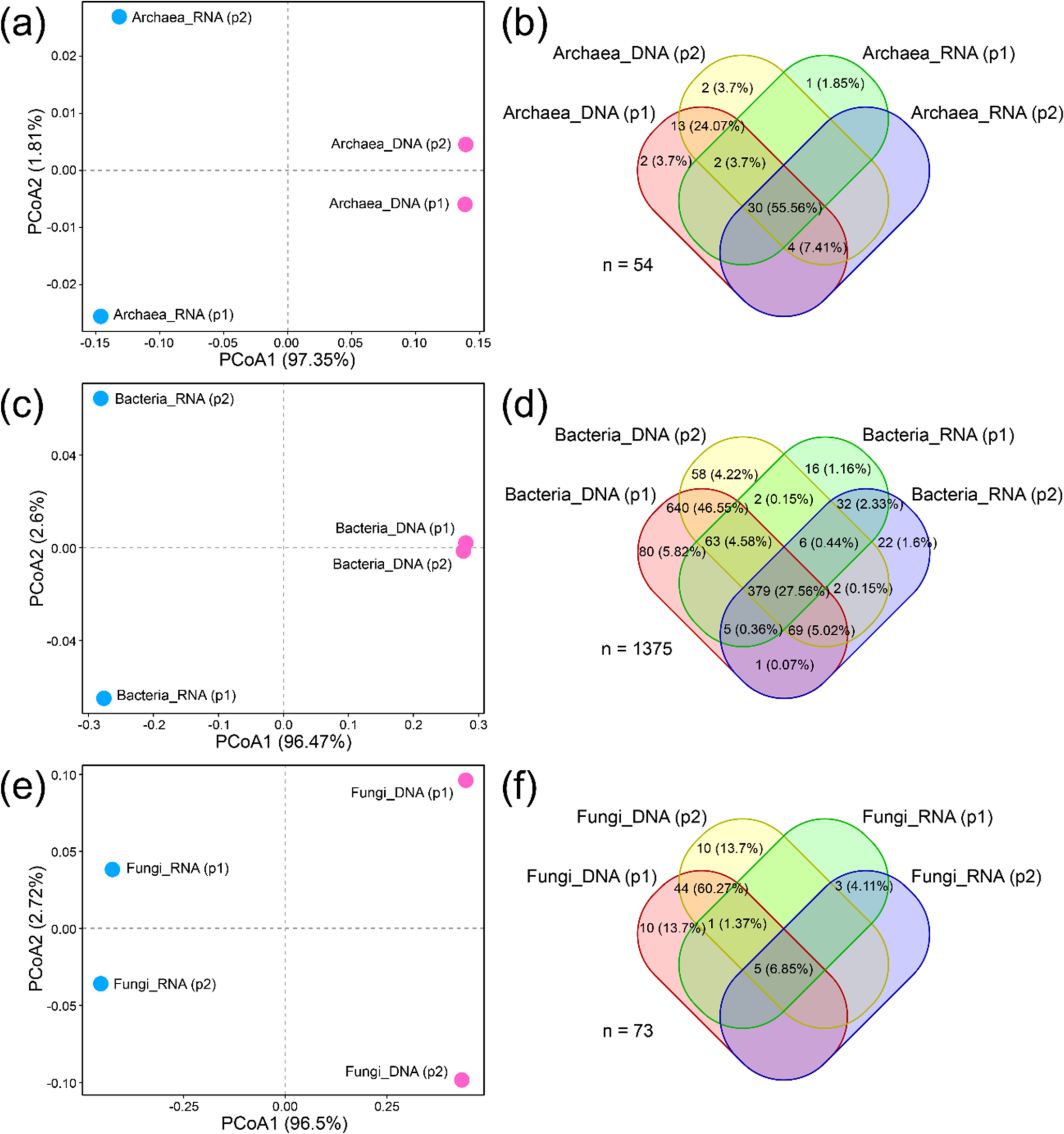
Beta diversity analysis, PCoA, based on Bray-Curtis dissimilarity, and Venn Diagram for analysis of sharedness and uniqueness of genera of the total/DNA and metabolically active/RNA microbial communities of (**a** and **b**) Archaea, (**c** and **d**) Bacteria, and (**e** and **f**) Fungi, from produced water samples from points p1 and p2. Sampling points: drain tank valve connected at 1.00 m height (p1) and 2.75 m height (p2). Average yield: biological replicate (BR) 1 and 2, each consisting of two technical replicates (TR)

Using metagenomic and metatranscriptomic sequences obtained from PW samples at points p1 and p2, the total/DNA and metabolically active/RNA microbial communities were assessed using Principal Coordinate Analysis (PcoA), based on Bray-Curtis dissimilarity (Fig. 6; Supplementary Material 5). Furthermore, the Venn diagram assessed the shared and unique genera among the samples (Fig. 6; Supplementary Material 5).

In the PCoA analyses, DNA and RNA PW samples from points p1 and p2 are in different quadrants (Fig. 6; Supplementary Material 5). PCoA1 and PCoA2 explain more than 98% of the total variability in the dataset of Archaea, Bacteria, and Fungi (Fig. 6). It is highlighted that the most significant variability is displayed on the PCoA1 axis (Fig. 6). The PCoA analysis was complemented by the PERMANOVA statistical test (*p* < 0.05), which evaluated the significance of dissimilarity among groups of total microbial communities/DNA and metabolically active/RNA for the Archaea, Bacteria, and Fungi of p1 and p2 (Fig. 6, Supplementary Material 5). Despite the results observed in the PCoA, there was no significant difference between the datasets for Archaea and Fungi (PERMANOVA, Archaea *p* = 0.0762; Fungi *p* = 0.0652), except for Bacteria (PERMANOVA, Bacteria *p* = 0.0494).

The total/DNA microbial communities for the Archaea, Bacteria, and Fungi from points p1 and p2 showed a higher absolute abundance of genera than active/RNA microbial communities (Fig. 6, Supplementary Material 5). In the Bacteria domain, a higher quantity was observed at both points p1 and p2, ranging from 1,219 to 1,237 and 503 to 516 genera for DNA and RNA samples, respectively (Fig. 6, Supplementary Material 5). Using metagenomic sequences, the Archaea exhibited 53 and Fungi 70 genera. In contrast, 37 (Archaeal) and 9 (fungal) genera were identified based on metatranscriptomic sequences (Fig. 6, Supplementary Material 5).

Among the total/DNA and active/RNA microbial communities from points p1 and p2, 30 shared genera of Archaea, 379 Bacteria, and 5 Fungi were identified (Fig. 6, Supplementary Material 5). There was the identification of unique Archaeal sequences belonging to the genera “Candidatus” *Prometheoarchaeum* and *Haloarcula* (DNA, p1), *Palaeococcus* and *Pyrococcus* (DNA, p2), and *Halolamina* (RNA, p1) (Fig. 6, Supplementary Material 5). In the analysis of fungal data, DNA sequences showed (p1 and p2) 10 unique genera. No unique genera at points p1 and p2 were observed in the RNA sequences of Fungi (Fig. 6, Supplementary Material 5). In contrast, in the analysis of Bacterial data, 16 to 80 unique genera were identified in the analyzed samples (Fig. 6, Supplementary Material 5).

### Functional profile of microbial communities

**Fig. 7:**
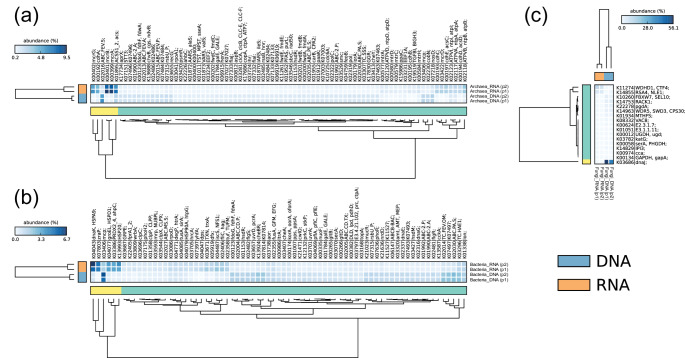

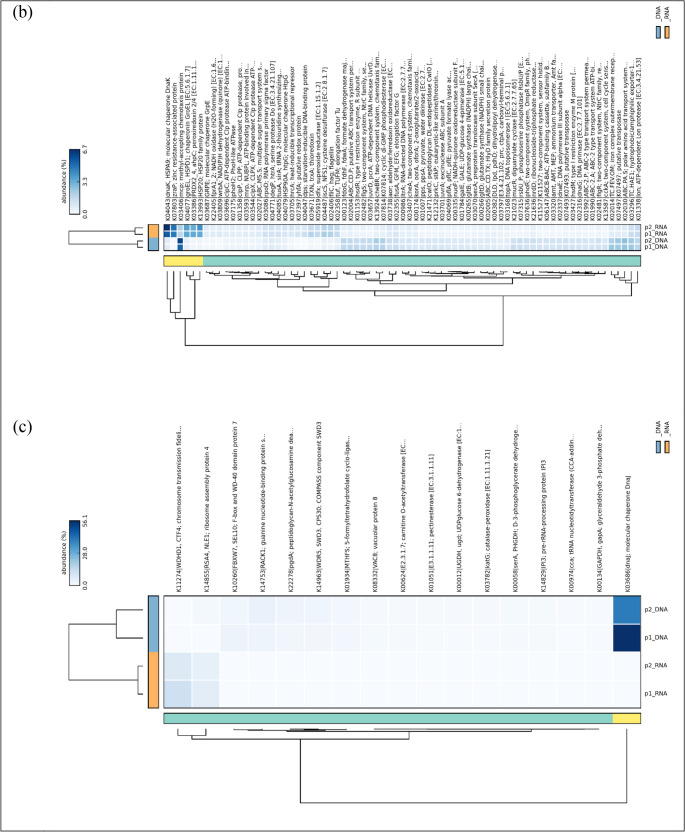
Functional Profile heatmaps used the matrices of the relative abundances of the 100 most abundant orthologous genes (KEGG ID) from the total/DNA and metabolically active/RNA microbial communities of (**a**) Archaea, (**b**) Bacteria, and (**c**) Fungi, from the produced water samples at points p1 and p2. Sampling points: drain tank valve connected at 1.00 m height (p1) and 2.75 m height (p2). Average yield: biological replicate (BR) 1 and 2, each consisting of two technical replicates (TR)

The heatmap of the functional profile predicted from the metagenomic and metatranscriptomic sequences highlighted the most abundant orthologous genes (KEGG ID) among the DNA and RNA samples from points p1 and p2 to Archaea, Bacteria, and Fungi (Fig. 7, Supplementary Material 6). These genes play crucial roles in the metabolic activities of the microbial communities. Notably, a disparity in the most abundant orthologous genes between DNA and RNA samples (p1 and p2) was observed related to the evaluated microbial groups (Fig. 7, Supplementary Material 6).

In the heatmap of the Archaea, the gene methyl-coenzyme M reductase alpha subunit (*mcrA*), beta subunit (*mcrB*), and gamma subunit (*mcrG*) exhibited higher abundance in RNA samples compared to DNA samples (Fig. 7, Supplementary Material 6). The gene anaerobic carbon-monoxide dehydrogenase, code/acs complex subunit alpha (*cdhA*), and, to a lesser extent, subunit gamma (*cdhE*), beta (*cdhC*), and delta (*cdhD*) also showed marked abundances in RNA samples from points p1 and p2 (Fig. 5). Additionally, in these same samples, the gene acetyl-CoA synthetase (*acss1_2 acs*) was among the most abundant (Fig. 7, Supplementary Material 6).

In the bacterial heatmap, the gene molecular chaperone Dnak (*dnak*, *hspa9*), zinc resistance-associated protein (*zrap*), chaperonin GroEL (*groEL*, *hspd1*), peroxiredoxin 2/4 (*prdx2_4*), and hsp20 family protein (*ahpc*, *hsp20*) were among the most abundant in RNA samples from points p1 and p2 (Fig. 7, Supplementary Material 6). On the other hand, the gene methyl-accepting chemotaxis protein (*mcp*) showed greater abundance in DNA samples (Fig. 7, Supplementary Material 6). When analyzing the heatmap of the Fungi, the gene molecular chaperone DnaJ (*dnaJ*) showed greater abundance in RNA samples from points p1 and p2 (Fig. 7, Supplementary Material 6).

## Discussion

Oil reservoirs contain high concentrations of hydrocarbons and often anoxic conditions (Li et al. 2017). Nevertheless, previous studies have shown that these ecosystems harbor both aerobic and anaerobic microbial communities, predominantly represented by Archaea and Bacteria (Shestakova et al. 2011; Nazina et al. 2017; Liu et al. 2018). These microorganisms play essential roles in hydrocarbon degradation and can influence microbially induced corrosion of pipelines, causing economic losses to the oil industry and environmental impacts on surrounding ecosystems. Therefore, understanding the diversity, composition, and functional profiles of these communities is critical for developing strategies to mitigate such risks (Liu et al. 2018; Salgar-Chaparro et al. 2020a, b; Salgar-Chaparro et al. 2020a, b b; Zhou et al. 2021; Zhou et al. 2022; Prajapat et al. 2023; Dutra et al. 2023).

In association with archaeal and bacterial communities, fungi also synthesize metabolic products, such as organic acids, during hydrocarbon biodegradation; thereby, contributing to biocorrosion processes (Beech and Gaylarde 1999; Zhang et al. 2017). Despite those relevant ecological functions, fungal communities in oil reservoirs and facilities remain underexplored.

Recently, Gomes et al. (2023) published a systematic review evaluating metabolically active microbial communities in produced water (PW) based on RNA sequencing. All studies analyzed focused exclusively on Archaea and Bacteria. Notably, the most commonly used method in these studies was 16 S rDNA and 16 S rRNA amplicon sequencing (metabarcoding), which restricts characterization to prokaryotes (Shestakova et al. 2011; Nazina et al. 2017; Li et al. 2017; Salgar-Chaparro and Machuca 2019; Zhou et al. 2020; Salgar-Chaparro et al. 2020a, b; Salgar-Chaparro et al. 2020a, b; Albahri et al. 2021). Even studies that applied metatranscriptomic analyses maintained their focus solely on Archaea and Bacteria (Liu et al. 2018, 2020a, b; Liu et al. 2020a, b).

Our study adopts a comprehensive approach, investigating the diversity, composition, and functional profiles of bacterial, archaeal, and fungal communities in Brazilian oil reservoirs. We employed high-throughput sequencing coupled to metagenomic and metatranscriptomic analyses, ensuring the depth and reliability of the data obtained. This integrated approach strengthens the robustness and originality of our findings.

Assessing alpha diversity, the total/DNA microbial communities of Archaea, Bacteria, and Fungi exhibited significantly higher taxonomic and functional diversity than the metabolically active/RNA communities (p1 and p2), a pattern similar to that reported by Li et al. (2017) for Archaea and Bacteria in PW, although other studies have observed variations among samples (Salgar-Chaparro and Machuca 2019; Zhou et al. 2020). This pattern can be explained by the fact that DNA-based analyses often show higher diversity values, as they recover genetic information from active, dormant, and dead cells. In contrast, RNA-based analyses recover information only from active cells (Salgar-Chaparro and Machuca 2019; Zhou et al. 2020), more accurately reflecting the contribution of active microbial communities to the various processes occurring in oil reservoirs and facilities.

In the total communities, the bacterial taxonomic (p1) and functional (p1 and p2) alpha diversity was statistically greater than that of Archaea and Fungi, with no significant difference between Archaea and Fungi (p2). Zhang et al. (2017) reported similar results in PW from shale gas reservoirs, whereas Guo et al. (2020) observed closer similarity between Bacteria and Fungi, indicating a strong influence of environmental context.

Among the metabolically active communities, Bacteria exhibited higher taxonomic and functional alpha diversity than Fungi, with no significant differences relative to Archaea. Li et al. (2017) likewise reported higher bacterial diversity in PW, whereas Zhou et al. (2020) observed that the most diverse community varied between Bacteria and Archaea depending on the sample. Not all studies; however, performed statistical validation of these comparisons (Li et al. 2017; Salgar-Chaparro and Machuca 2019; Zhou et al. 2020).

In other analyses, the metabolically active/RNA archaeal communities at p1 and p2 were primarily associated with the environmental variables pH, SO₄²⁻, S²⁻, salinity, electrical conductivity (EC), and dissolved solids (DS), correlating with hydrogenotrophic methanogens (*Methanocorpusculum*, *Methanoregula*, *Methanocalculus*, *Methanoplanus*) and methylotrophs (*Methanolobus*), since these variables modulate the availability and utilization of H₂, CO₂, and methylated compounds; thereby, favoring methanogenic pathways (Hartman et al. 2024). Conversely, the total/DNA archaeal communities showed stronger relationships with acetate, alkalinity, butyrate, Cl⁻, Fe, and turbidity, being associated with aceticlastic (*Methanothrix*), hydrogenotrophic (*Methanothermobacter*, *Methanococcus*), and methylotrophic (*Methanohalophilus*) genera. Those parameters reflect both the direct availability of substrates (acetate) and indirect generation via butyrate fermentation, as well as environmental conditions (alkalinity, salts, and iron) that modulate pH stability, osmolarity, and syntrophic processes releasing H₂, favoring methanogenic activity (Nazaries et al. 2013; Conrad 2020).

It is noteworthy that in both the DNA and RNA samples from p1 and p2 we observed a predominance of the genera *Methanocalculus*, *Methanoplanus*, and *Methanothrix* within the phylum Methanobacteriota, mirroring patterns observed in systematic reviews of metabolically active microorganisms in reservoir fluids (Gomes et al. 2023). *Methanocalculus* (mesophilic) utilizes hydrogen and formate, whereas *Methanothrix* (thermophilic) depends on acetate during anaerobic degradation of organic matter (Kamagata et al. 1992; Nazina et al. 2017), indicating that distinct methanogenic routes are favored by PW geochemical gradients. Consistently, previous studies have also reported these genera in PW and injection water from oil fields (Nazina et al. 2017; Salgar-Chaparro and Machuca 2019; Zhou et al. 2020).

At the functional level, analyses of archaeal communities revealed high abundance of the *mcrABDG* genes in RNA samples from p1 and p2; these encode methyl-coenzyme M reductase (MCR), a key enzyme that catalyzes the final step of methanogenesis and the initial step of anaerobic methane oxidation (Gendron and Allen 2022; Zhou et al. 2022). Variants of MCR have also been implicated in the anaerobic oxidation of short-chain alkanes, including ethane, propane, and butane, as well as in the catabolism of long-chain alkanes to CO₂ (Gendron and Allen 2022; Zhou et al. 2022). The *mcrA* gene, in particular, has been widely used as a biomarker to identify total and active methanogenic populations in archaeal communities from oil reservoirs (Liu et al. 2020a, b; Liu et al. 2020a, b; Zhou et al. 2021; Gomes et al. 2023). Moreover, detection of *acs* and *cdhABCD* genes in RNA samples indicates activation of the aceticlastic pathway, reinforcing the role of acetate as a relevant substrate for methanogenesis under PW conditions (Liu et al. 2018; Do Amaral et al. 2019).

For bacteria, the analyses revealed a distinction between the total and active communities at p1 and p2; notably, the PW at p2 is located closer to the oil phase, which favors the predominance of genera specialized in hydrocarbon metabolism and syntrophic interactions (Dutra et al. 2023), without excluding the occurrence of the same metabolic functions at p1.

At p1, bacterial community clusters were primarily associated with the environmental variables pH, SO₄²⁻, S²⁻, salinity, electrical conductivity (EC), and dissolved solids (DS), correlating with sulfate reducers (*Desulfobacter*), syntrophs (*Syntrophotalea* and *Mesotoga*), and anaerobic fermenters (*Sphaerochaeta*) in DNA samples, as well as *Desulfuromonas* in RNA samples, which is metabolically active, reduces metal oxides and sulfur, and is potentially capable of hydrocarbon degradation. Those patterns suggest that those variables modulate the availability of electrons and substrates (such as H₂, formate, and sulfur compounds), favoring the activity of microorganisms involved in anaerobic respiration and syntrophic interactions (Rabus et al. 2013; Schink and Stams 2013). At p2, bacterial communities were more strongly related to acetate, alkalinity, butyrate, Cl⁻, Fe, and turbidity, being represented by sulfate-reducing genera (*Pseudodesulfovibrio*), hydrocarbon degraders and syntrophs (*Geotoga* and “Candidatus” *Cloacimonas*) in DNA samples, and by other sulfate reducers (*Oceanidesulfovibrio* and *Paucidesulfovibrio*) and a halophilic fermenter (*Halanaerobium*) in RNA samples. These parameters reflect both the direct availability of substrates (acetate, hydrocarbons) and indirect generation via butyrate fermentation, as well as environmental conditions (alkalinity, salts, iron) that modulate pH stability, osmolarity, and syntrophic processes that release H₂; thereby, favoring anaerobic, sulfate-reducing bacterial activity (Sorokin et al. 2008; Muyzer and Stams 2008; Rabus et al. 2013; Schink and Stams 2013; Zhang et al. 2022).

In the total/DNA bacterial communities, the genera *Syntrophotalea* and *Pseudodesulfovibrio* (phylum Pseudomonadota), and in the metabolically active/RNA communities the genus *Halanaerobium* (phylum Bacillota), were the most abundant. These microorganisms are strictly anaerobic and perform essential metabolic functions, such as sulfate reduction, hydrocarbon degradation, acid production, and syntrophy (Pereira et al. 2021; Gaikwad et al. 2023), processes directly related to microbially influenced corrosion in oil infrastructure (Li et al. 2017; Salgar-Chaparro and Machuca 2019). According to Gomes et al. (2023), both phyla are among the most representative metabolically active microorganisms in PW samples.

Functional analyses of the bacterial communities are consistent with the observed patterns. In RNA samples from p1 and p2, genes related to cellular stress (*dnaK*-*hspA9*, *zraP*, *groEL*-*hspD1*, *prdx2*_4-*ahpC*, and *hsp20*) were highly abundant, evidencing active bacterial responses to adverse conditions. Although sulfate-reducing bacteria were relatively abundant in the taxonomic profiles, genes directly involved in sulfate reduction (*aprAB* and *dsrABC*) showed low expression, suggesting that other metabolic routes and stress-response mechanisms predominate at the transcriptional level. The high proportion of “unclassified” taxa indicates activity of poorly characterized groups that cannot be assigned to known genera.

Fungal communities, including decomposers and fermentative yeasts, showed associations with the environmental variables analyzed. At p1, the activity of the metabolically active/RNA communities was related to pH, SO₄²⁻, S²⁻, salinity, EC, and DS, reflecting the predominance of genera with different metabolic capacities, such as decomposers and acid producers (*Penicillium*, *Rhizopus*, *Saccharomycodes*). At p2, fungal composition was influenced by dissolved organic compounds, such as acetate and butyrate, favoring genera specialized in hydrocarbon degradation (*Absidia*) and fermentation (*Pichia*, *Schizosaccharomyces*, and *Hyphopichia*) in both total/DNA and active/RNA communities.

Previous studies have isolated fungal genera such as *Absidia*, *Rhizopus*, *Penicillium*, and *Neopestalotiopsis* from fluids and sludges in the oil industry, demonstrating potential for the remediation of soils and effluents contaminated with polycyclic aromatic hydrocarbons (PAHs), BTEX, and other recalcitrant compounds (Xie et al. 2016; Aranda et al. 2017; Usman et al. 2020; Guo et al. 2020; Fallahi et al. 2023; Zhou et al. 2023; Mohammed et al. 2023). Those fungi utilize hydrocarbons as energy sources and produce biosurfactants, increasing substrate availability for other Fungi, Bacteria, and Archaea, and influencing the physicochemical properties of oil, potentially benefiting microbial enhanced oil recovery (MEOR) processes (Othman et al. 2022; Zhang et al. 2024).

In PW, the phylum Mucoromycota dominated in the total/DNA communities, whereas Ascomycota dominated the metabolically active/RNA communities. Among the most relevant genera, *Absidia* and *Penicillium* exhibit the capacity to degrade hydrocarbons and recalcitrant compounds (Wolski 2023), whereas *Saccharomycodes*, *Pichia*, and *Schizosaccharomyces* stand out for their fermentative activity and adaptation to toxin-rich environments, potentially acting synergistically with Bacteria and Archaea in hydrocarbon degradation (Xie et al. 2016; Othman et al. 2022).

Functional analysis revealed a high abundance of the *dnaJ* gene in RNA samples from both points, indicating that fungal communities are metabolically active and adapted to cellular stress conditions, maintaining proteostasis via the DnaK/DnaJ/GrpE system (Kumar et al. 2022). These genes help fungi survive harsh petroleum environments, maintaining protein homeostasis under chemical and thermal stress. Functionally, those fungi contribute to biosurfactant production, increased bioavailability of insoluble compounds, and potential biogas generation factors that may enhance microbial mobilization and recovery of oil (MEOR) (Zhang et al. 2024). Furthermore, the metabolic activities of these fungi, including acid production, biofilm formation, and degradation of hydrocarbons, may directly influence corrosion processes in petroleum environments by altering metal surfaces and local chemistry, creating conditions that favor microbially influenced corrosion. This mechanistic link highlights the potential role of active fungal communities in both ecological functions and industrially relevant deterioration of infrastructure. Considering the absence of prior studies evaluating active fungal communities in PW, the data obtained provide a pioneering perspective on the dynamics, functions, and both ecological and industrial relevance of those microorganisms in oil reservoirs.

Our findings demonstrate that, in oil reservoirs, the structure and activity of archaeal, bacterial, and fungal communities are strongly modulated by geochemical gradients and substrate availability, reflecting distinct metabolic strategies and syntrophic interactions that sustain hydrocarbon degradation and processes associated with biocorrosion, highlighting the complementary and underexplored role of metabolically active fungal communities. These insights provide a valuable basis for practical applications in reservoir management, enabling the development of targeted strategies to mitigate MIC in pipelines, a critical economic and environmental concern (Li et al. 2017; Salgar-Chaparro and Machuca 2019). Furthermore, the identification of metabolically active sulfate-reducing bacterial genera, methanogenic Archaea, and fermentative or hydrocarbon-degrading fungi allows for specific interventions, such as controlled biocide application (Zhang et al. 2017; Othman et al. 2022). Notably, knowledge of active fungal genera and their metabolic roles can support bioremediation strategies and microbial enhanced oil recovery processes, increasing hydrocarbon mobilization while limiting the impact of corrosive microbial activity (Xie et al. 2016; Zhang et al. 2024). In this way, our study not only characterizes microbial diversity but also establishes the foundation for future research and industrial practices aimed at monitoring, managing, and harnessing microbial activities in oil reservoirs.

## Conclusion

High-throughput DNA and RNA sequencing, coupled with metagenomic and metatranscriptomic analyses, allowed a comprehensive characterization of Archaea, Bacteria, and Fungi in PW samples. Total/DNA communities displayed greater diversity than metabolically active/RNA communities, indicating that only a subset of genera and their associated metabolic activities are actively engaged. Bacteria exhibited significantly higher diversity than Archaea and Fungi, consistent with previous studies on PW microbiota in oil reservoirs. Importantly, this study also revealed the presence of metabolically active, hydrocarbon-degrading Fungi, highlighting their previously underexplored ecological role. These findings underscore the need for further research into fungal activity and their interactions with Archaea and Bacteria, which will be a focus of future investigations.

## Supplementary Information

Below is the link to the electronic supplementary material.

Supplementary Material 1

Supplementary Material 2

Supplementary Material 3

Supplementary Material 4

Supplementary Material 5

Supplementary Material 6

## Data Availability

All metagenomic and metatranscriptomic sequences are available in the NCBI Sequence Read Archive (SRA) under accession number PRJNA1029995.
