# Supplementary material for: Metagenomics and metatranscriptomics of prokaryotic and fungal microbiomes in produced water associated with petroleum degradation and pipeline corrosion from an oil terminal in Brazil
Source: World J Microbiol Biotechnol. 2026 Jun 17;42(7):357. doi: 10.1007/s11274-026-05012-x (PMC13275535; doi:10.1007/s11274-026-05012-x)
Supplement: Supplementary file 1 — Supplementary Material 1 [file 11274_2026_5012_MOESM1_ESM.docx]

**SUPPLEMENTARY MATERIAL 1.** **Physicochemical characterization of produced water samples collected at sampling points p1 and p2. (Dutra et al., 2023).**

| **Feature** | **Unit** | **Collection points** | |
| --- | --- | --- | --- |
|  |  | **p1** | **p2** |
| pH | **-** | 7.00 | 6.00 |
| Lactate | mg/L | n.d | n.d |
| Acetate | mg/L | 970.00 | 1500.00 |
| Propionate | mg/L | n.d | n.d |
| Formate | mg/L | n.d | n.d |
| Butyrate | mg/L | 29.00 | 67.00 |
| Sulfate | mg/L | 310.00 | 170.00 |
| Soluble sulfides | mg/L | 57.80 | 54.90 |
| Chloride | mg/L | 2.60 | 2.80 |
| Iron | mg/L | 0.48 | 0.59 |
| Alkalinity*^,1^ | meqs/L | 25.50 | 28.70 |
| Salinity^2^ | ppt | 37.50 | 36.40 |
| Electrical conductivity^3^ | mS | 65.00 | 60.71 |
| Dissolved solids | mg/L | 31.55 | 30.25 |
| Turbidity^4^ | NTU | 90.13 | 106.25 |

Sampling points: drain tank valve connected at 1.00 m height (p1) and 2.75 m height (p2). nd: not detectable. *1st inflection: pH = 3.2 (p1), pH = 3.0 (p2). Unit: ^1^milliequivalents per liter (megs/L); ^2^parts per thousand (ppt); ^3^Milisiemens (mS); ^4^**Nephelometric turbidity unit (NTU)**
