# Supplementary material for: Metagenomics and metatranscriptomics of prokaryotic and fungal microbiomes in produced water associated with petroleum degradation and pipeline corrosion from an oil terminal in Brazil
Source: World J Microbiol Biotechnol. 2026 Jun 17;42(7):357. doi: 10.1007/s11274-026-05012-x (PMC13275535; doi:10.1007/s11274-026-05012-x)
Supplement: Supplementary file 2 — Supplementary Material 2 [file 11274_2026_5012_MOESM2_ESM.docx]

**SUPPLEMENTARY MATERIAL 2.** **Yields** **and purity of total DNA and RNA extracted from filters containing retained biomass of produced water samples collected at sampling points p1 and p2.**

| **Sample** | **Replicate** | **Concentration (ng/µl)** | | | | | **Yield (ng)** | | |  |  | **A_260/280_** | | | | | **A_260/230_** | | | | |
| --- | --- | --- | --- | --- | --- | --- | --- | --- | --- | --- | --- | --- | --- | --- | --- | --- | --- | --- | --- | --- | --- |
|  |  | **BR1** | **BR2** | **Media** | | **SD** | **BR1** | **BR2** | **Média** |  | **SD** | **BR1** | **BR2** | **Media** | | **SD** | **BR1** | **BR2** | **Media** | | **SD** |
| **DNA (p1)** | TR1 | 306,10 | 247,70 | 276,90 | ± | 29,20 | 24488,00 | 19816,00 | 22.152,00 | ± | 2.336,00 | 1,86 | 1,85 | 1,86 | ± | 0,01 | 0,27 | 0,2 | 0,26 | ± | 0,02 |
|  | TR2 | 269,60 | 222,20 | 245,90 | ± | 23,70 | 21568,00 | 17776,00 | 19.672,00 | ± | 1.896,00 | 1,85 | 1,85 | 1,85 | ± | 0,00 | 0,21 | 0,2 | 0,20 | ± | 0,01 |
|  | **Media** | **287,85** | **234,95** | **261,40** | **±** | **26,45** | **23028,00** | **18796,00** | **20.912,00** | **±** | **2.116,00** | **1,86** | **1,85** | **1,85** | **±** | **0,00** | **0,24** | **0,22** | **0,23** | **±** | **0,01** |
| **DNA (p2)** | TR1 | 319,20 | 284,60 | 301,90 | ± | 17,30 | 25536,00 | 22768,00 | 24.152,00 | ± | 1.384,00 | 1,83 | 1,85 | 1,84 | ± | 0,01 | 0,25 | 0,5 | 0,37 | ± | 0,12 |
|  | TR2 | 329,20 | 277,00 | 303,10 | ± | 26,10 | 26336,00 | 22160,00 | 24.248,00 | ± | 2.088,00 | 1,84 | 1,84 | 1,84 | ± | 0,00 | 0,42 | 0,30 | 0,36 | ± | 0,06 |
|  | **Media** | **324,20** | **280,80** | **302,50** | **±** | **21,70** | **25936,00** | **22464,00** | **24.200,00** | **±** | 1.736,00 | **1,84** | **1,85** | **1,84** | **±** | **0,01** | **0,34** | **0,39** | **0,36** | **±** | **0,09** |
| **RNA (p1)** | TR1 | 438,50 | 563,50 | 501,00 | ± | 62,50 | 35080,00 | 45080,00 | 40.080,00 | ± | 5.000,00 | 1,93 | 1,73 | 1,83 | ± | 0,10 | 0,64 | 0,39 | 0,52 | ± | 0,13 |
|  | TR2 | 417,00 | 648,70 | 532,85 | ± | 115,85 | 33360,00 | 51896,00 | 42.628,00 | ± | 9.268,00 | 1,92 | 1,66 | 1,79 | ± | 0,13 | 0,60 | 0,30 | 0,45 | ± | 0,15 |
|  | **Media** | **427,75** | **606,10** | **516,93** | **±** | **89,18** | **34220,00** | **48488,00** | **41.354,00** | **±** | **7.134,00** | **1,93** | **1,70** | **1,81** | **±** | **0,12** | **0,62** | **0,35** | **0,48** | **±** | **0,14** |
| **RNA (p2)** | TR1 | 441,10 | 931,70 | 686,40 | ± | 245,30 | 35288,00 | 74536,00 | 54.912,00 | ± | 19.624,00 | 1,88 | 1,77 | 1,83 | ± | 0,05 | 0,6 | 0,43 | 0,50 | ± | 0,07 |
|  | TR2 | 168,20 | 753,20 | 460,70 | ± | 292,50 | 13456,00 | 60256,00 | 36.856,00 | ± | 23.400,00 | 1,60 | 1,82 | 1,71 | ± | 0,11 | 0,2 | 0,47 | 0,36 | ± | 0,12 |
|  | **Media** | **304,65** | **842,45** | **573,55** | **±** | **268,90** | **24372,00** | **67396,00** | **45.884,00** | **±** | **21.512,00** | **1,74** | **1,80** | **1,77** | **±** | **0,08** | **0,40** | **0,45** | **0,43** | **±** | **0,09** |

Sampling points: drain tank valve connected at 1.00 m height (p1) and 2.75 m height (p2).

BR: Biological replicate 1 and 2. Each BR is composed of two technical replicates.

**Figure 1. DNA and RNA yield extracted from filters containing retained biomass of produced water samples collected at sampling points p1 and p2, considering two biological replicates. BR: biological replicate 1 and 2. Each BR consists of two technical replicates.**
