# Supplementary material for: Metagenomics and metatranscriptomics of prokaryotic and fungal microbiomes in produced water associated with petroleum degradation and pipeline corrosion from an oil terminal in Brazil
Source: World J Microbiol Biotechnol. 2026 Jun 17;42(7):357. doi: 10.1007/s11274-026-05012-x (PMC13275535; doi:10.1007/s11274-026-05012-x)
Supplement: Supplementary file 3 — Supplementary Material 3 [file 11274_2026_5012_MOESM3_ESM.docx]

**SUPPLEMENTARY MATERIAL 3. Shannon, Chao1, Evenness and Simpson indices, at the genus level, of the total/DNA and metabolically active/RNA microbial communities of archaea, bacteria, and fungi, from the produced water samples at points p1 and p2.**

| **Sample\| Index** | **Shannon for taxonomy** | | | | | **Shannon for functional** | | | | | **Chao1 for taxonomy** | | | | | | **Evenness for taxonomy** | | | | | | **Simpson for taxonomy** | | | | | |
| --- | --- | --- | --- | --- | --- | --- | --- | --- | --- | --- | --- | --- | --- | --- | --- | --- | --- | --- | --- | --- | --- | --- | --- | --- | --- | --- | --- | --- |
|  | **BR1** | **BR2** | **Media** |  | **SD** | **BR1** | **BR2** | **Media** |  | **SD** | **BR1** | **BR2** | **Media** |  | **SD** | **BR1** | | **BR2** | **Media** |  | **SD** | **BR1** | | **BR2** | **Media** |  | **SD** |  |
| Archaea_DNA (p1) | 2,281 | 2,282 | 2,282 | **±** | 0,001 | 7,031 | 7,038 | 7,035 | **±** | 0,003 | 174,111 | 170,125 | 172,118 | **±** | 1,993 | 0,442 | | 0,444 | 0,443 | **±** | 0,001 | 0,858 | | 0,848 | 0,853 | **±** | 0,005 |  |
| Bacteria_DNA (p1) | 3,393 | 3,439 | 3,416 | **±** | 0,023 | 7,662 | 7,674 | 7,668 | **±** | 0,006 | 2125,735 | 2127,902 | 2126,819 | **±** | 1,083 | 0,443 | | 0,449 | 0,446 | **±** | 0,003 | 0,938 | | 0,942 | 0,940 | **±** | 0,002 |  |
| Fungi_DNA (p1) | 2,627 | 2,702 | 2,664 | **±** | 0,038 | 2,085 | 2,251 | 2,168 | **±** | 0,083 | 66,526 | 60,375 | 63,451 | **±** | 3,076 | 0,627 | | 0,660 | 0,643 | **±** | 0,016 | 0,853 | | 0,870 | 0,862 | **±** | 0,009 |  |
| Archaea_DNA (p2) | 2,114 | 2,317 | 2,215 | **±** | 0,101 | 7,030 | 7,067 | 7,048 | **±** | 0,018 | 166,231 | 176,000 | 171,115 | **±** | 4,885 | 0,413 | | 0,448 | 0,431 | **±** | 0,017 | 0,802 | | 0,854 | 0,828 | **±** | 0,026 |  |
| Bacteria_DNA (p2) | 3,430 | 3,430 | 3,430 | **±** | 0,000 | 7,673 | 7,670 | 7,672 | **±** | 0,001 | 2084,156 | 2103,089 | 2093,622 | **±** | 9,467 | 0,449 | | 0,448 | 0,449 | **±** | 0,000 | 0,946 | | 0,945 | 0,945 | **±** | 0,000 |  |
| Fungi_DNA (p2) | 2,190 | 2,781 | 2,485 | **±** | 0,296 | 2,535 | 2,440 | 2,488 | **±** | 0,047 | 66,929 | 59,667 | 63,298 | **±** | 3,631 | 0,529 | | 0,682 | 0,605 | **±** | 0,077 | 0,724 | | 0,881 | 0,803 | **±** | 0,078 |  |
| Archaea_RNA (p1) | 2,147 | 2,329 | 2,238 | **±** | 0,091 | 5,399 | 6,673 | 6,036 | **±** | 0,637 | 102,000 | 117,000 | 109,500 | **±** | 7,500 | 0,464 | | 0,489 | 0,477 | **±** | 0,012 | 0,807 | | 0,854 | 0,831 | **±** | 0,024 |  |
| Bacteria_RNA (p1) | 3,195 | 3,386 | 3,291 | **±** | 0,095 | 6,896 | 7,400 | 7,148 | **±** | 0,252 | 840,497 | 1004,984 | 922,741 | **±** | 82,244 | 0,475 | | 0,490 | 0,482 | **±** | 0,008 | 0,927 | | 0,941 | 0,934 | **±** | 0,007 |  |
| Fungi_RNA (p1) | 1,336 | 0,534 | 0,935 | **±** | 0,401 | 1,271 | 1,778 | 1,525 | **±** | 0,254 | 6,000 | 9,000 | 7,500 | **±** | 1,500 | 0,745 | | 0,243 | 0,494 | **±** | 0,251 | 0,630 | | 0,193 | 0,412 | **±** | 0,219 |  |
| Archaea_RNA (p2) | 2,178 | 2,238 | 2,208 | **±** | 0,030 | 5,812 | 6,685 | 6,249 | **±** | 0,436 | 95,000 | 121,000 | 108,000 | **±** | 13,000 | 0,478 | | 0,467 | 0,472 | **±** | 0,006 | 0,812 | | 0,840 | 0,826 | **±** | 0,014 |  |
| Bacteria_RNA (p2) | 3,072 | 3,305 | 3,188 | **±** | 0,117 | 6,925 | 7,272 | 7,099 | **±** | 0,174 | 739,751 | 1059,940 | 899,845 | **±** | 160,094 | 0,465 | | 0,475 | 0,470 | **±** | 0,005 | 0,913 | | 0,934 | 0,924 | **±** | 0,011 |  |
| Fungi_RNA (p2) | 0,636 | 1,439 | 1,037 | **±** | 0,401 | 1,074 | 2,091 | 1,583 | **±** | 0,509 | 7,000 | 11,000 | 9,000 | **±** | 2,000 | 0,327 | | 0,600 | 0,463 | **±** | 0,137 | 0,263 | | 0,585 | 0,424 | **±** | 0,161 |  |

Sampling points: tank valve connected at 1.00 m height (p1) and 2.75 m height (p2). BR: Biological replica 1 and 2.
